# Ultrasound-Assisted Minimally Invasive Clavicle Plate Removal

**DOI:** 10.7759/cureus.98593

**Published:** 2025-12-06

**Authors:** Sreenivasulu Metikala, Dominique B Spence, Cesar Cardenas, Robert Blackstone, Naga Cheppalli

**Affiliations:** 1 Orthopaedic Foot & Ankle Surgery, Veterans Affairs Medical Center, Richmond, USA; 2 Orthopaedic Surgery, Virginia Commonwealth University School of Medicine, Richmond, USA; 3 Orthopaedics and Rehabilitation, University of New Mexico School of Medicine, Albuquerque, USA; 4 Orthopaedic Surgery, University of New Mexico School of Medicine, Albuquerque, USA; 5 Orthopaedic Surgery, Raymond G. Murphy Veterans Affairs Medical Center, Albuquerque, USA; 6 Orthopaedics, Veterans Affairs (VA) Hospital Albuquerque, Albuquerque, USA

**Keywords:** clavicle fracture, clavicle osteosynthesis, clavicle plate, clavicle plate removal, point-of-care-ultrasound

## Abstract

We present the feasibility of point-of-care ultrasound (POCUS) to aid minimally invasive surgery (MIS) for clavicular plate removal. We present a case of a 29-year-old male who sustained a displaced midshaft clavicle fracture after a bike accident. On day four, post-injury, he underwent an open reduction and internal fixation with an anatomic 3.5mm locking plate, complicated by numbness of the anterior chest extending to the medial arm. Four years post-operatively, he presented for hardware irritation, which was palpable on physical exam. After unsuccessful conservative treatments, the patient elected to undergo hardware removal utilizing POCUS as the mode for a minimally invasive approach. The MIS approach, known for reducing wound complications and nerve damage, is traditionally confirmed using a C-arm image intensifier. POCUS, being a portable and non-ionizing imaging modality, serves as an alternative to traditional methods. POCUS allows for a more precise surgical incision that is smaller than the traditional open approach, with the additional advantage of visualization of neurovascular structures compared to a C-arm. Post-operatively, the patient’s course was uncomplicated with no surgical site numbness and participation in previous activities at six weeks. Although additional training is required to become an expert in acquiring ultrasound skills, most hardware can be easily located through ultrasound.

## Introduction

Hardware irritation is a common problem after clavicle plate osteosynthesis, often necessitating implant removal with a reported incidence of nine to 44% [1]. Traditionally, clavicle plate removal is done by an open approach requiring exploration through previously operated tissue planes. The open surgical approach entails longer incisions and dissection through the scar tissues with subsequent morbidity, such as wound-healing complications, anterior chest wall numbness, and decreased shoulder range of motion (ROM). On the other hand, the minimally invasive surgical (MIS) approach for removing hardware may result in a lower rate of surgical complications, thus optimizing early functional outcomes [2]. Traditionally, a C-arm image intensifier is utilized for the MIS approach to assist in placing surgical incisions directly over the screw heads during this approach. As is well known, the C-arm poses radiation risk and mandates a technician, as well as spacious operating suites [3]. The alternative tool is a point-of-care ultrasound (POCUS), a radiation-free imaging modality that can localize the underlying hardware and also the adjacent neurological structures, facilitating safe surgical removal. In the present case study, we describe the application of POCUS for the MIS technique of the clavicle plate. 

## Case presentation

**Figure 1:**
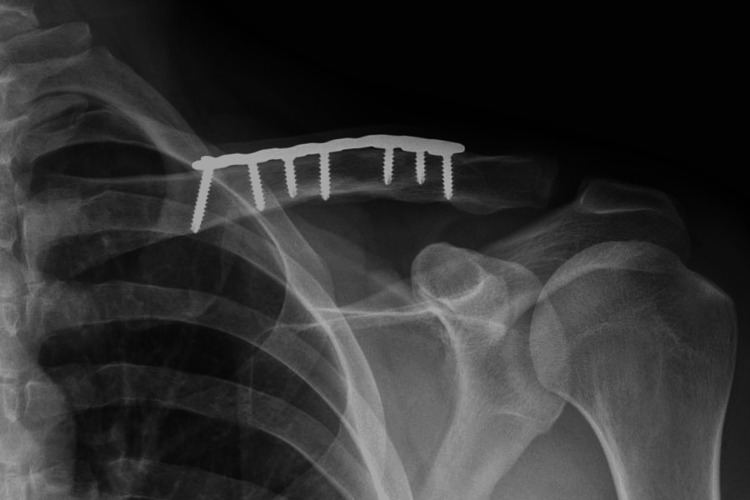
Postoperative radiograph showing a healed fracture in anatomic alignment after osteosynthesis using a superior plate fixated by seven screws.

A 29-year-old male sustained a displaced midshaft clavicle fracture of the nondominant left arm (Figure 1) following a bike accident and underwent an open reduction and internal fixation (ORIF) on day four post-injury. An 8-holed anatomical 3.5 mm locking Plate (Smith & Nephew EVOS small plating system, TN, USA) was utilized along the superior surface, fixated with seven screws. The fracture healed about three months postoperatively, and the patient returned to his previous activities. However, he subsequently developed symptoms of hardware irritation, with frequent pain and palpable hardware at the surgical incision. He presented to us four years after the index clavicle ORIF with a keen interest in hardware removal. The clinical examination showed a 10 cm surgical scar healed by primary intention with tender and prominent hardware. There were no inflammatory signs or limitation of adjacent shoulder motion, and he was neurovascularly intact. The radiographs (Figure 1) demonstrated a healed midshaft clavicle fracture with the maintenance of alignment and stable hardware position. The initial conservative treatment with activity modifications and over-the-counter pain medicines did not improve his symptoms, so the patient elected for hardware removal. After a thorough preoperative discussion, we employed an MIS approach using a POCUS device to assist with the intraoperative localization of hardware.

**Figure 2:**
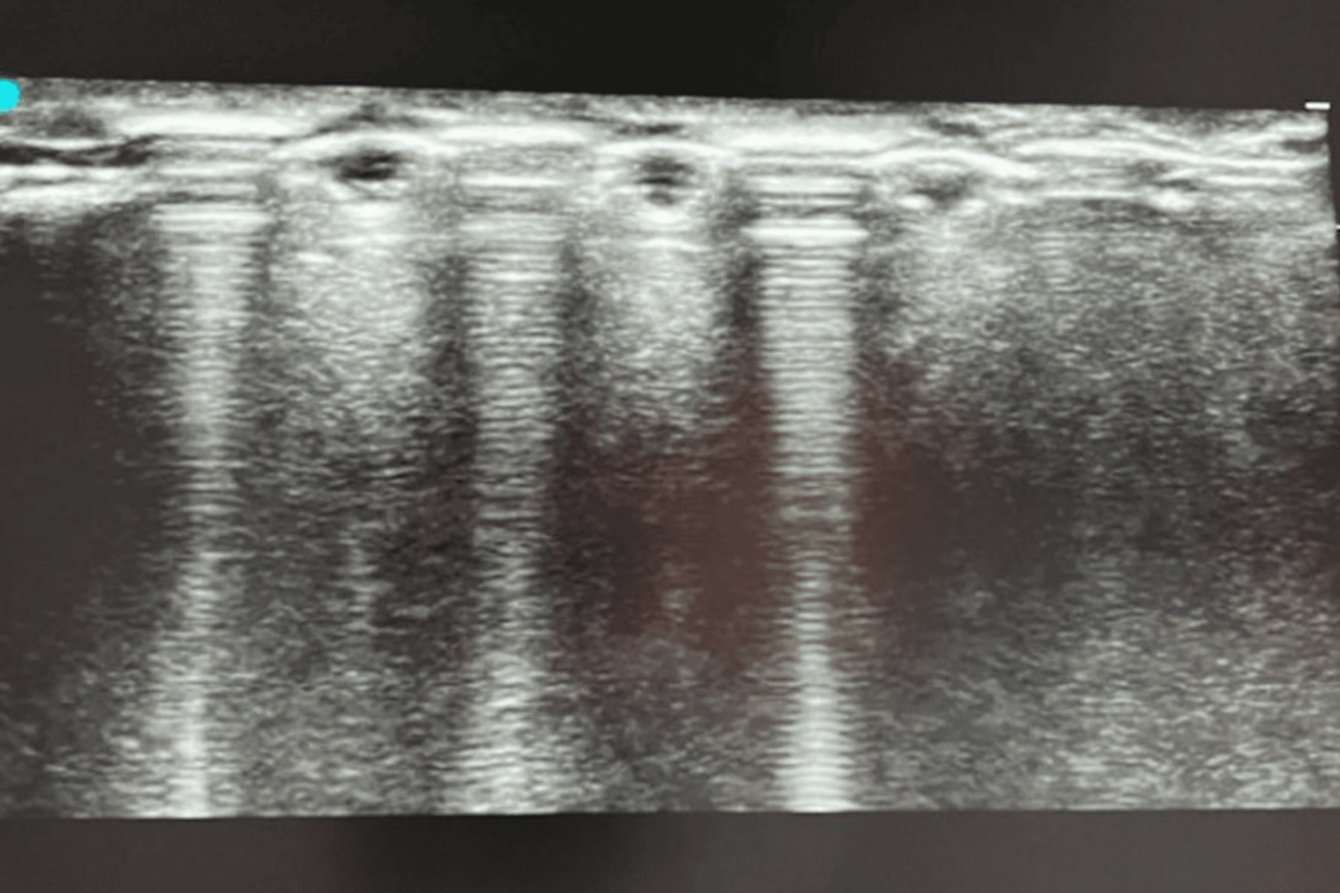
Ultrasonographic visualization of screws and plate.

**Figure 3:**
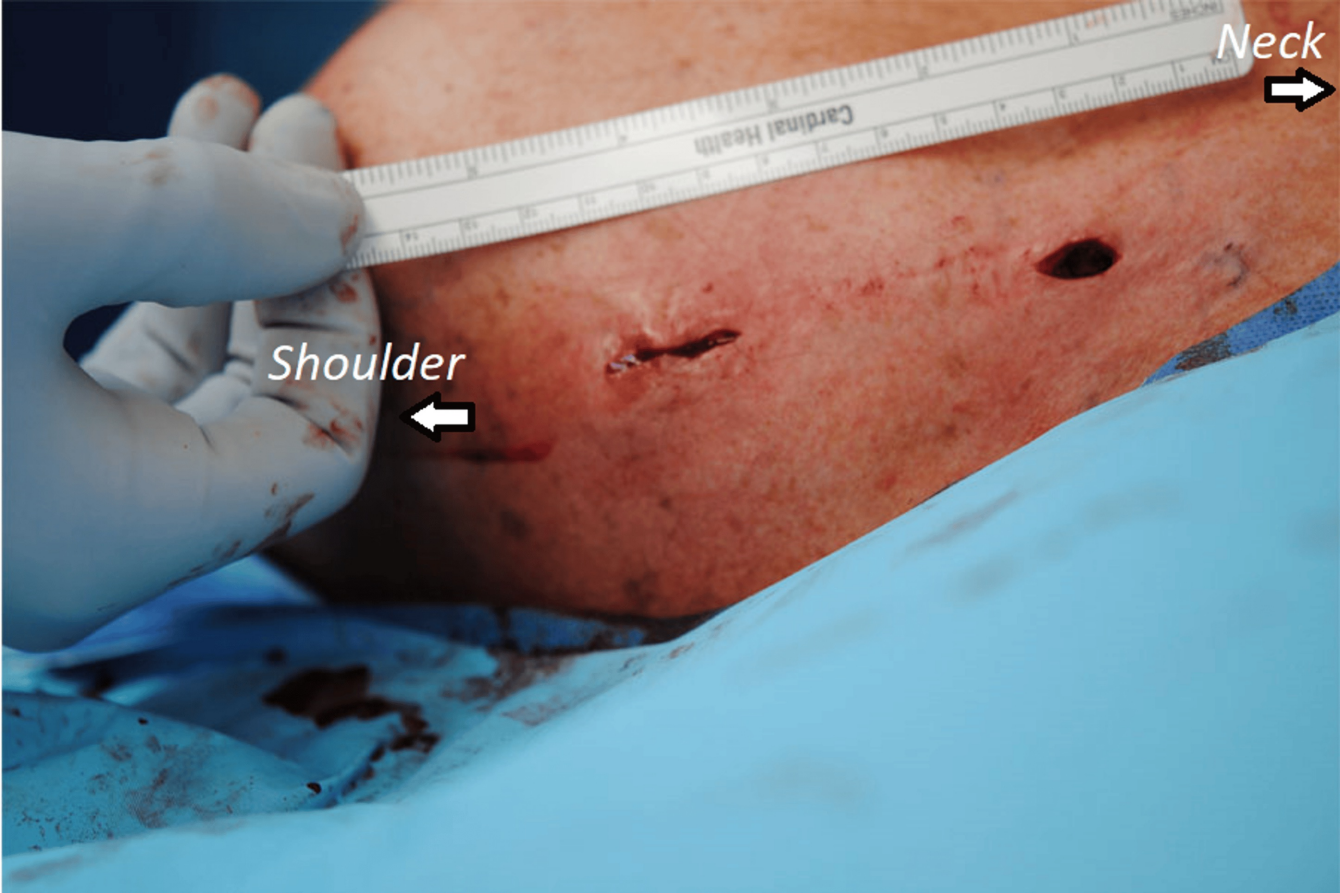
Intraoperative image with the patient in supine and 30-degree head elevation showing the placement of two skin incisions for MIS removal of hardware from the left clavicle.

The surgery was performed under general anesthesia with the patient in the supine position and 30° head elevation. A safety timeout was performed, followed by prepping and draping of the surgical field in a standard sterile fashion. A high-frequency linear transducer with a sterile wrap was employed in longitudinal and transverse planes, identifying individual screws as a hyperechogenic structure with reverberation artifact (Figure 2). Two 1.5-cm incisions were marked on either side of the previous scar, and the skin was incised directly over the screw heads (Figure 3). With meticulous subcutaneous dissection, mobile skin windows were created to access all the screw heads, which were cleared off the soft tissues and removed. A thin osteotome was then slid under the plate, freeing the adhesions with the underlying clavicle and removing the plate from the lateral window by extending the skin incision. The surgical wounds were lavaged and closed in a layered fashion.

Postoperative course

**Figure 4:**
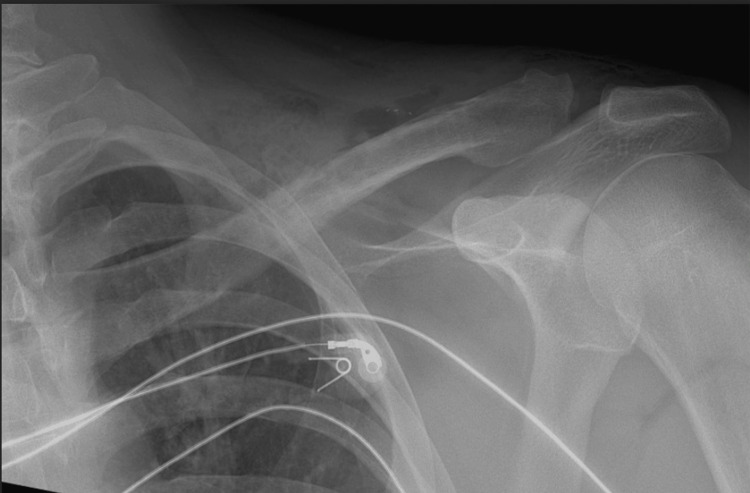
Post-implant removal radiograph showing no retained hardware.

The immediate post-operative radiographs (Figure 4) confirmed the complete hardware removal. Postoperatively, the patient was encouraged to immediately regain shoulder ROM, as pain allowed, and the surgical sutures were removed at two weeks. At six weeks, the patient reported resolution of previous symptoms of hardware irritation with no tenderness at the surgical site and mild numbness in the anterior chest. He returned to his previous activities and was discharged from our follow-up after three months.

After his index procedure, the patient reported significant numbness in the anterior chest region extending up to the nipple, medially up to the sternum, and laterally up to the middle third of the arm. Over the three years before clavicular plate removal, the numbness gradually resolved. During the explant procedure, the patient noted that the area of numbness was approximately 10% to 15% less compared to their index surgery, and the numbness was not as profound as before. Furthermore, the patient reported that their symptoms gradually resolved over a period of three months. Quick dash score (patient-reported outcome) is 2.5 and has no pain with some numbness along the anterior chest wall, significantly lower than when he had clavicle osteosynthesis.

## Discussion

In this study, we presented the technical feasibility of an ultrasound-assisted MIS approach for plate removal from the clavicle. Unlike the conventional open technique, the MIS approach obviates the need to fully re-explore the previous surgical incision and underlying scarred tissue planes. Therefore, soft tissue damage and the resultant complications, including hypertrophic scar, wound infection, and infraclavicular numbness, are minimized. The nerve-related complications, such as anterior chest wall numbness with dysesthesia, have a reported incidence of 12-29% [4]. These occur due to supraclavicular nerve injury and frequently contribute to patient dissatisfaction postoperatively, which may be preventable with the MIS approach [5]. 

Although POCUS is not new to Orthopedic surgeons, it is mainly limited to the office environment for evaluating tendon/ligament injuries and administering steroid injections. Regarding hardware removal, the C-arm Image intensifier has been a favorite intraoperative imaging modality, but is not routinely used. This practice can be attributed to the lack of formal ultrasound education during residency training or published literature in orthopedic journals. As opposed to the C-arm, POCUS offers several advantages. It is readily available in most operating rooms as anesthesiologists frequently use it for peripheral nerve blocks. It is a non-ionizing and portable apparatus that surgeons can use in a sterile field by placing the probe in a sterile wrap [6]. The metallic object, such as the screw head in this reported case, exhibits a typical hyperechoic appearance with reverberation artifact [6]. As a consequence, it is quite straightforward to diagnose, even for early adopters. Where applicable, the surrounding neurovascular structures can also be readily identified by using the device in Doppler mode, permitting safe surgical extraction of the hardware. The surgical incision can be planned precisely by placing the initial skin cut just over one of the central screws. With minimal subcutaneous dissection, this incision can then be swayed on either side as a mobile window to access the rest of the screws for removal. The only limitation, however, is the requirement of formal ultrasound training and practice, as it is a user-dependent modality. Proficiency in bedside ultrasonography has become a standard inclusion in the emergency medicine (EM) residency curriculum. On the contrary, formal ultrasound education has not yet been introduced into the orthopaedic residency curriculum, although the learning curve is not huge for orthopedic applications [7].

## Conclusions

The commonality of clavicular plate irritation necessitates utilizing minimally invasive approaches for symptomatic hardware removal. We have shown that a POCUS can be used intraoperatively for clavicle plate removal by a MIS approach. This approach allows for greater surgical precision and visualization of neurovascular structures to reduce the risk of postoperative numbness, infection, and hypertrophic scarring. Unlike a C-arm, it is a portable and non-ionizing tool for orthopedic surgeons, but it requires formal training with a learning curve to acquire adequate skills. 

## References

[1] Hulsmans MH, van Heijl M, Houwert RM (2017). High irritation and removal rates after plate or nail fixation in patients with displaced midshaft clavicle fractures. Clin Orthop Relat Res.

[2] Beirer M, Postl L, Crönlein M (2015). Does a minimal invasive approach reduce anterior chest wall numbness and postoperative pain in plate fixation of clavicle fractures?. BMC Musculoskelet Disord.

[3] Hayda RA, Hsu RY, DePasse JM, Gil JA (2018). Radiation exposure and health risks for orthopaedic surgeons. J Am Acad Orthop Surg.

[4] Christensen TJ, Horwitz DS, Kubiak EN (2014). Natural history of anterior chest wall numbness after plating of clavicle fractures: educating patients. J Orthop Trauma.

[5] Jiang H, Qu W (2012). Operative treatment of clavicle midshaft fractures using a locking compression plate: comparison between mini-invasive plate osteosynthesis (MIPPO) technique and conventional open reduction. Orthop Traumatol Surg Res.

[6] Del Cura JL, Aza I, Zabala RM, Sarabia M, Korta I (2020). US-guided localization and removal of soft-tissue foreign bodies. Radiographics.

[7] Blehar DJ, Barton B, Gaspari RJ (2015). Learning curves in emergency ultrasound education. Acad Emerg Med.

